# AGE LOGICS IN SOCIAL WORK: THE CASE OF HARM REDUCTION FOR 50+ WITH LONG-TERM SUBSTANCE MISUSE

**DOI:** 10.1093/geroni/igad104.2319

**Published:** 2023-12-21

**Authors:** Håkan Jönson, Tove Harnett

**Affiliations:** Lunds Universitet, Lund, Skane Lan, Sweden; Lund University, Lund, Skane Lan, Sweden

## Abstract

Age is a commonly used criterion in social work: for entry and exit and in decisions on what actions being appropriate for different clients. This study introduces the concept of age logics in social work and do so by investigating the use of age in wet eldercare facilities, a type of harm reduction arrangements for older people. These settings are for people over 50 years of age with long term substance misuse. No treatment is provided, and residents can continue to consume alcohol and other substances at the facilities for the rest of their lives. Using wet eldercare facilities as a case, the aim of this article is: 1) to introduce age logics as an analytical tool for critical studies on age in social work and 2) to propose a method for how to reveal ageism embedded in locally used age logics. The empirical data consists of interviews with eleven caseworkers, 12 staff-members and 31 residents at two Swedish wet eldercare facilities. The analysis identified four age logics linking chronological age with four meanings: 1) Logic on changeability, 2) Logic on lifestyle 3) Logic on function and 4) Logic on administrative fit. Jointly these logics constructed an ideal type version of the “older addict” that justified existing arrangements. The analysis also served as a basis for a proposal of a “four-question method” that may be used to reveal and challenge ageism hidden in locally used age logics.

